# Supplementary material for: A Multi-Level Analysis of Individual and Neighborhood Factors Associated with Patient Portal Use among Adult Emergency Department Patients with Multimorbidity
Source: Int J Environ Res Public Health. 2023 Jan 10;20(2):1231. doi: 10.3390/ijerph20021231 (PMC9859180; doi:10.3390/ijerph20021231)
Supplement: Supplementary file 1 [file ijerph-20-01231-s001.zip › ijerph-2058370-supplementary.pdf]

Supplementary Materials:

Table S1. Correlation analyses of Different Community-level Variables.

	Couple	Citizen	Employ	Income	Insurance	Poverty	Vehicle	Multi-race	Language	Hispanic	Education	Internet
Couple	1.00											
Citizen	-0.4570	1.00										
Employ	-0.5671	0.0988	1.00									
Income	0.6948	-0.5724	-0.5297	1.00								
Insurance	-0.3246	0.7608	0.0968	-0.6813	1.00							
Poverty	-0.7656	0.5697	0.6532	-0.7579	0.5436	1.00						
Vehicle	-0.5461	0.0998	0.4984	0.4084	-0.0429	0.6534	1.00					
Multi-race	0.0371	-0.2451	-0.2920	0.2673	-0.3305	-0.2869	0.0006	1.00				
Language	-0.3376	0.5216	0.1963	-0.5838	0.6979	0.5877	0.3260	-0.0513	1.00			
Hispanic	-0.2695	0.5753	0.1889	-0.5800	0.7588	0.5555	0.2274	-0.1533	0.9548	1.00		
Education	0.3443	-0.4823	-0.4747	0.8562	-0.6945	-0.5783	-0.2018	0.4159	-0.5308	-0.5974	1.00	
Internet	0.6742	-0.5848	-0.5765	0.7937	-0.6497	-0.8889	-0.5017	0.3704	-0.5430	-0.5590	0.6993	1.00

Table S2. Patient Portal Use among Adult ED Patient with Multimorbidity Distributed by Different Insurance Coverages.

Insurance Coverage	Patients with Portal Use	Patients without Portal Use
Hospital Sponsored Insurance	2,877 (31%)	6,354 (69%)
No Insurance Coverage	675 (7%)	8,804 (93%)
Other types of Insurance	4,205 (19%)	17,629 (81%)

$P<0.0001$.
